# Loss in MCL-1 function sensitizes non-Hodgkin's lymphoma cell lines to the BCL-2-selective inhibitor venetoclax (ABT-199)

**DOI:** 10.1038/bcj.2015.88

**Published:** 2015-11-13

**Authors:** D C Phillips, Y Xiao, L T Lam, E Litvinovich, L Roberts-Rapp, A J Souers, J D Leverson

**Affiliations:** 1 Oncology Discovery, AbbVie Inc., North Chicago, IL, USA; 2 Abbott Molecular, Des Plaines, IL, USA; 3 Oncology Development, AbbVie Inc., North Chicago, IL, USA

## Abstract

As a population, non-Hodgkin's lymphoma (NHL) cell lines positive for the t(14;18) translocation and/or possessing elevated *BCL2* copy number (CN; *BCL2*^*High*^) are exquisitely sensitive to navitoclax or the B-cell lymphoma protein-2 (BCL-2)-selective inhibitor venetoclax. Despite this, some *BCL2*^*High*^ cell lines remain resistant to either agent. Here we show that the MCL-1-specific inhibitor A-1210477 sensitizes these cell lines to navitoclax. Chemical segregation of this synergy with the BCL-2-selective inhibitor venetoclax or BCL-X_L_-selective inhibitor A-1155463 indicated that MCL-1 and BCL-2 are the two key anti-apoptotic targets for sensitization. Similarly, the CDK inhibitor flavopiridol downregulated MCL-1 expression and synergized with venetoclax in *BCL2*^*High*^ NHL cell lines to a similar extent as A-1210477. A-1210477 also synergized with navitoclax in the majority of *BCL2*^*Low*^ NHL cell lines. However, chemical segregation with venetoclax or A-1155463 revealed that synergy was driven by BCL-X_L_ inhibition in this population. Collectively these data emphasize that *BCL2* status is predictive of venetoclax potency in NHL not only as a single agent, but also in the adjuvant setting with anti-tumorigenic agents that inhibit MCL-1 function. These studies also potentially identify a patient population (*BCL2*^*Low*^) that could benefit from BCL-X_L_ (navitoclax)-driven combination therapy.

## Introduction

Apoptosis or programmed cell death is an evolutionarily conserved cellular process that is required for normal embryonic development and maintenance of tissue homeostasis. The B-cell lymphoma protein-2 (BCL-2) family of proteins are essential regulators of apoptosis, functioning as either activators or inhibitors of cell death primarily at the mitochondrial level. This family of proteins consists of three groups that each contain at least one BCL-2 homology (BH) motif (BH1-4). The pro-apoptotic ‘BH3-only' proteins, BID, BIM, PUMA, NOXA, BAD, BIK, BMF and HRK are activated or induced by cell death stimuli that, in turn, may activate the pro-apoptotic ‘multidomain effector' proteins BAX and BAK. Once activated, these proteins homo-oligomerize to induce mitochondrial outer membrane permeabilization. Mitochondrial outer membrane permeabilization results in the release of pro-apoptotic factors such as cytochrome *c* from the mitochondria into the cytosol leading to apoptosome formation, caspase activation and DNA fragmentation. The anti-apoptotic members (BCL-2, BCL-X_L_, MCL-1, BCL-W and BFL-1) contain multiple BH motifs and function to inhibit apoptosis by direct interaction with the ‘BH3-only' and multi-domain effectors via their BH3-binding grooves. Aberrant expression and/or function of BCL-2 family members results in deregulation of apoptosis that contributes to the development of a variety of human pathologies including cancer, neurodegeneration and autoimmunity.^1, 2^

Non-Hodgkin's lymphoma (NHL) represents a heterogeneous group of lymphoid-derived malignancies that include follicular lymphoma, diffuse large B-cell lymphoma and mantle cell lymphoma (MCL). The t(14;18) chromosomal translocation results in *BCL2* hyperexpression by juxtaposing it to the immunoglobulin heavy chain gene enhancer, representing the primary tumorigenic event in most follicular lymphomas that is also found in ~20% of diffuse large B-cell lymphomas.^3, 4^ Elevated expression of BCL-2 in diffuse large B-cell lymphoma is also associated with *BCL2* gene amplification or transcriptional upregulation through constitutive activation of the NFκB pathway.^5, 6^ BCL-2 overexpression is associated with poor prognosis^5, 7^ in NHL by promoting cell survival and resistance to anti-tumorigenic agents.^1, 2, 8^ Transgenic mouse models also reveal that MCL-1 and BCL-X_L_ hyperexpression contribute to the onset and maintenance of hematological malignancies.^9, 10, 11, 12^

Navitoclax (ABT-263) is an orally bioavailable anti-tumorigenic agent that targets BCL-2, BCL-X_L_ and BCL-W but not MCL-1 or BFL-1^(ref. 13)^ and is being evaluated in clinical trials as a single agent or in the adjuvant setting. However, BCL-X_L_-driven thrombocytopenia has been dose limiting in patients with hematological malignancies or small cell lung cancer.^14, 15, 16, 17, 18, 19^ Consequently, we developed the BCL-2-selective inhibitor venetoclax (ABT-199) that shows superior affinity to BCL-2 relative to navitoclax and circumvents BCL-X_L_-driven thrombocytopenia.^20^ This attribute may permit attainment of higher plasma concentrations that translate into improved response rates in patients with BCL-2-dependent malignancies. Despite this, some cell lines of hematologic origin remain resistant to both venetoclax and navitoclax.^20^

Although *BCL2* is frequently mutated in NHL,^21, 22^ these mutations do not affect sensitivity to ABT-737^(ref. 22)^ and are unlikely to affect navitoclax or venetoclax efficacy. Mutations have been described in murine *BCL2* following ABT-737/venetoclax acquired resistance,^23^ however the analogous mutations in human *BCL2* have not been reported in NHL patients. Therefore, potential inherent resistance factors may reside elsewhere in the apoptotic pathway. For example, MCL-1 has been identified by us and numerous other investigators as a factor that contributes to both intrinsic and acquired resistance to ABT-737, navitoclax and venetoclax.^24, 25, 26, 27, 28^ Merino *et al.*^29^ have suggested that navitoclax is not an efficient antagonist of BCL-X_L_ in lymphoid cells, indicating that BCL-X_L_ is in fact a resistance factor for ABT-737^(refs 29, 30)^ and potentially navitoclax as well as, more obviously, venetoclax. Using highly potent and selective inhibitors of BCL-2,^(ref. 20)^ BCL-X_L_^(ref. 31)^ or MCL-1,^(refs 27, 28, 32)^ and combinations thereof, we sought to further classify the survival dependency of NHL for anti-apoptotic BCL-2 family members. Consequently, these pre-clinical data inform on strategies to potentially improve on the clinical efficacy of venetoclax through co-inhibition of MCL-1 function.

## Materials and methods

### Reagents, cell culture and treatment

NHL cell lines were obtained from the American Type Culture Collection or Deutsche Sammlung von Mikroorganismen und Zellkulturen and were cultured in Iscove's Modified Dulbecco's Media containing 10% human serum and 10 mM L-glutamine (all from Invitrogen Corporation, Carlsbad, CA, USA). All cell lines were tested for authenticity by short tandem repeat profiling and mycoplasm by the AbbVie Core Cell Line Facility. Cells were plated at a density of 0.25 × 10^6^cells/well in six-well plates for apoptosis assays, at 0.1 × 10^6^/ml for cell viability assays, and at 3 × 10^6^per 10 cm^2^ petri dish for western blots. Navitoclax, venetoclax, A-1210477 and A-1155463 were dissolved in anhydrous dimethyl sulfoxide to a stock solution of 10 mM. Flavopiridol was dissolved in dimethyl sulfoxide at 1 mM. After overnight attachment, cells were treated for up to 48 h with vehicle alone, navitoclax, venetoclax, A-1155463, flavopiridol or A-1210477, or in the described combinations. Where indicated, cells were pre-treated for 60 min with z-VAD-fmk (50 μM; MP Biomedicals, Santa Ana, CA, USA). Navitoclax, venetoclax, A-1155463 and A-1210477 were synthesized as described.^20, 31, 32, 33^ Unless otherwise indicated, all chemical reagents were obtained from Sigma Aldrich (St. Louis, MO, USA).

### Cell viability

Cells (0.1 × 10^6^/ml) were treated in 96-well plates for 72 h and cell viability determined by CellTiter-Glo as described by the manufacturer's instructions (Promega Corporation, Madison, WI, USA). Responses were determined as a percentage of the control treated cells and EC_50_s determined from sigmoidal dose-response curves using GraphPad Prism (GraphPad Software, La Jolla, CA, USA).

### Annexin-V/7-AAD staining

Apoptosis was determined by flow cytometric evaluation of Annexin-V/7-AAD staining as described in detail elsewhere.^34^

### Western blot analysis

After treatment, cells were washed twice with ice-cold PBS containing 10% fetal bovine serum, centrifuged at 1000 r.p.m. for 5 min, and lysed in 50 μl of ice-cold Cell Lytic (Sigma) supplemented with protease (Roche Diagnostics Corporation, Indianapolis, IN, USA) and phosphatase (Sigma) inhibitors. Protein concentrations were determined by the BSA assay (Invitrogen) and 50 μg of protein electrophoresed by SDS–PAGE (Invitrogen). Separated proteins were transferred to nitrocellulose membranes utilizing an iBlot (Invitrogen) device. Blots were probed with MCL-1 (clone S-19; Santa Cruz Biotechnology, La Jolla, CA, USA), PARP (clone C2-10) and BCL-2 (Clone 7; both BD Biosciences, CA, USA), caspase-3 (clone 31A1067, Abcam, Cambridge, UK) or β-actin (Sigma) antibodies followed by IRDye 680/800CW-conjugated secondary antibodies (LICOR Biosciences, NE, USA). Proteins were visualized using the Odyssey infrared imaging system (LICOR Biosciences) and were not further manipulated with imaging software.

### Fluorescent *in situ* hybridization (FISH)

PBS-washed cells (2–3 × 10^6^ cells/ml) were isolated on BioGenex dual spot barrier slides (100 μl per spot) for 5 min at 500 r.p.m. before fixation with 1% formaldehyde. Slides were washed twice in PBS, air dried and stored at 4 °C before FISH. FISH was performed using a custom protocol on a Biogenex Xmatrx automated staining instrument. Briefly, slides underwent cell dehydration with ethanol, heat denaturation (96 ^o^C, 5 min) and incubation with Vysis LSI IgH:*BCL2* translocation fusion probe set (Abbott Molecular Diagnostics, 05J71-001) at 42 ^o^C for 14 h, followed by a stringency wash with 2X SSC, and application of 4′,6-diamidino-2-phenylindole to stain nuclei. The IgH:*BCL2* translocation status was then determined by fluorescence microscopy at × 100 magnification (Zeiss AxioPhot 2 fluorescence microscope; Oberkochen, Germany).

### Determination of *BCL2, BCL2L1* and *MCL1* CN

DNA was isolated from NHL cell lines using DNeasy blood and tissue kit (Qiagen, Venlo, Netherlands; #69506) per manufacturer's protocol, except eluted in reduced EDTA TE buffer (Teknova, Hollister, CA, USA; T0223) and quantitated with PicoGreen assay (Molecular Probes, Thermo-Fisher, Waltham, MA, USA). Copy number was determined by SNP 6.0 assay (500 ng DNA input) per manufacture's protocol (Affymetrix cytogenetics copy number assay rev. 2) followed by data smoothing and quantitation of CEL files in Partek software (Partek Inc., St Louis, MO, USA).

### Protein expression

BCL-2, BCL-X_L_ and MCL-1 protein expression were measured using an assay developed based on the Luminex technology (Austin, TX, USA). In brief, MCL-1, BCL-2 and BCL-X_L_ capture antibodies were custom conjugated to Luminex carboxyl beads (bead region 9, 33 and 64, respectively) by Millipore (St. Charles, MO, USA). MCL-1 detection antibody was also conjugated to biotin through a custom service provided by Millipore. BCL-2 and BCL-X_L_ detection antibodies conjugated to biotin were included in the DuoSetIC kits from R&D Systems (Minneapolis, MN, USA). Cells were lysed in MILLIPLEX MAP lysis buffer 1 (Millipore Cat. no. 43-040, Danvers, MA, USA) containing protease inhibitor cocktail (Sigma). Protein expression was determined using a Luminex FlexMap 3D system (Luminex) as described in depth elsewhere.^35^ Data are presented as median fluorescent intensity.

### Electrochemiluminescent ELISA

Streptavidin multi-array 96-well plates (Meso Scale Discovery (MSD), Gaithersburg, MD, USA) were used to immobilize biotin-labeled anti-BCL-2 (US Biological, catalog no. B0807-067), biotin-labeled anti-BCL-X_L_ (Abcam, catalog no. ab25062), biotin-labeled anti-MCL-1 (NeoMarker, catalog MS-681-B) and biotin-labeled IgG1 (US Biological, catalog no. 11904-6A2). Protein samples (75 μg; extracted with CHAPS buffer containing protease and phosphatase inhibitors; Roche and Sigma, respectively), were subsequently added to each plate in duplicate. The plate was incubated overnight at 4 °C to pull down BCL-2. After three washes with PBS-tween, anti-BIM (Epitomics; catalog no. 1036-1) was added and incubated for 1 h in the dark at room temperature with rotation at 650 r.p.m. Subsequently, sulfo-tagged goat anti-rabbit antibody (MSD; Rockville, MD, USA) was added to each well and incubated for a further 30 min as mentioned above and then washed three times with PBS-tween. Finally, 150 μl of 2 × MSD read buffer T was added per well and fluorescence measured with a MSD Sector Imager 6000 (MSD, Gaithersburg, MD, USA).

### Statistical analysis

Data are represented as the mean±s.e.m. In all cases, the number of independent experiments is described within the figure legend. The Mann–Whitney *U*-test was used to determine statistical significance. Spearman's rank correlation co-efficient was used to determine statistical dependence between two variables. The Bliss independence model was used to evaluate synergy.^36^

## Results

**Figure 1:**
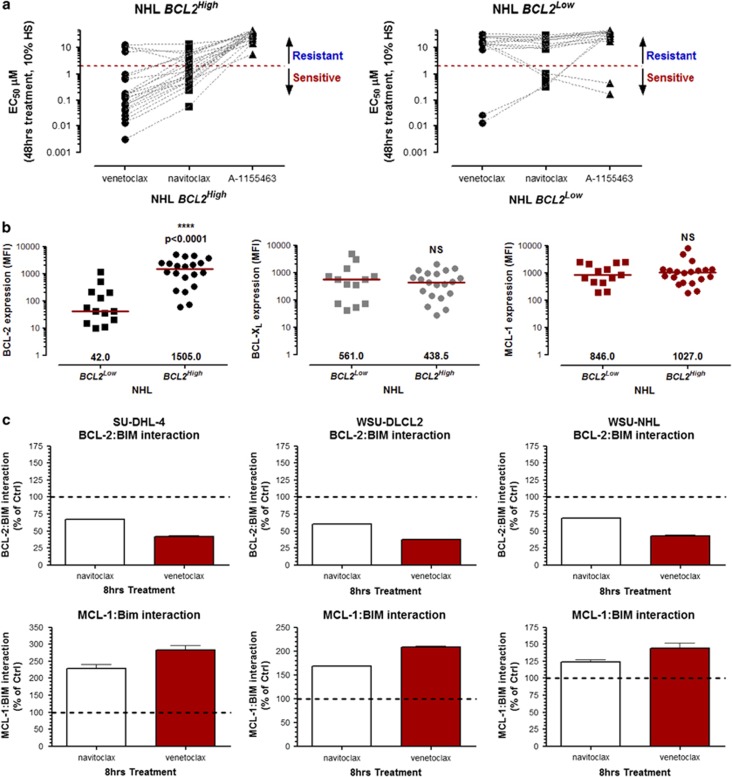
Chemical segregation of navitoclax activity in NHL cell lines; requirement for BCL-2 or BCL-X_L_ for survival. The efficacy of navitoclax, venetoclax or A-1155463 was determined in NHL cell lines as described in the Materials and Methods section. EC_50_s were calculated from the resulting sigmoidal dose/response curves (see Supplementary Table 1) and segregated according to their *BCL2*^*High*^ or *BCL2*^*Low*^ status.^20^ Data are presented as the mean of at least three independent experiments. Cell lines with navitoclax EC_50_s >2 μM were deemed resistant. Data are presented as the mean of at least three independent experiments (**a**). Expression of anti-apoptotic BCL-2 family proteins was determined by Luminex as described and segregated according to the *BCL2*^*High*^ or *BCL2*^*Low*^ status. The median is shown in red and the Mann–Whitney *U*-test was used to determine statistical significance. NS, not significant (**b**). Navitoclax-resistant *BCL2*^*High*^ cells were treated with navitoclax or venetoclax (both 1 μM) for 8 h and the interaction of BIM with BCL-2 or MCL-1 assessed using an Electrochemiluminescent ELISA (MSD) as described in the Materials and Methods section. Data are presented as the mean±s.e.m. of three independent experiments (**c**).

We recently described the BCL-2-selective inhibitor venetoclax to show superior potency to navitoclax in pre-clinical models of hematological cancers. Venetoclax *in vitro* potency correlates with the expression of BCL-2 in NHL cell lines. Furthermore, segregation of NHL cell lines into *BCL2*^*High*^ (t(14;18)^+^ and/or high *BCL2* CN) and *BCL2*^*Low*^ groups identifies the former group as being particularly sensitive to venetoclax.^20^ Here we have characterized additional NHL cell lines for sensitivity to venetoclax and navitoclax as well as their CN and/or t(14;18) translocation status (Supplementary Table 1). To assess the contribution of BCL-X_L_ for survival, we treated all NHL cell lines with BCL-X_L_-selective inhibitor A-1155463.^31^ As expected, *BCL2*^*High*^ cell lines were resistant to the BCL-X_L_-selective inhibitor A-1155463 (EC_50_ >5 μM). Although the majority of *BCL2*^*Low*^ NHL cell lines were also resistant to navitoclax, SU-DHL-8 and RCK8 were sensitive. Importantly, navitoclax sensitivity in SU-DHL-8 and RCK8 was driven by BCL-X_L_ inhibition since both cell lines were sensitive to A-1155463 and resistant to venetoclax (Figure 1a and Supplementary Figure 1). We next assessed protein expression of the anti-apoptotic BCL-2 family members in these NHL cell lines using the Luminex FlexMap 3D system.^35^ As expected, BCL-2 protein expression was significantly higher in *BCL2*^*High*^ cell lines relative to *BCL2*^*Low*^ cell lines. However, MCL-1 and BCL-X_L_ protein levels were approximately the same in either population (Figure 1b). Furthermore, the BCL-X_L_ protein expression in the A-1155463-sensitive cell lines RCK8 and SU-DHL-8 was 4721 and 719 median fluorescent intensity, respectively (Figure 1b), and did not reflect the EC_50_ of A-1155463 in each cell line (446.4 nM and 167.4 nM, respectively; Figure 1a and Supplementary Figure 1).

**Figure 2:**
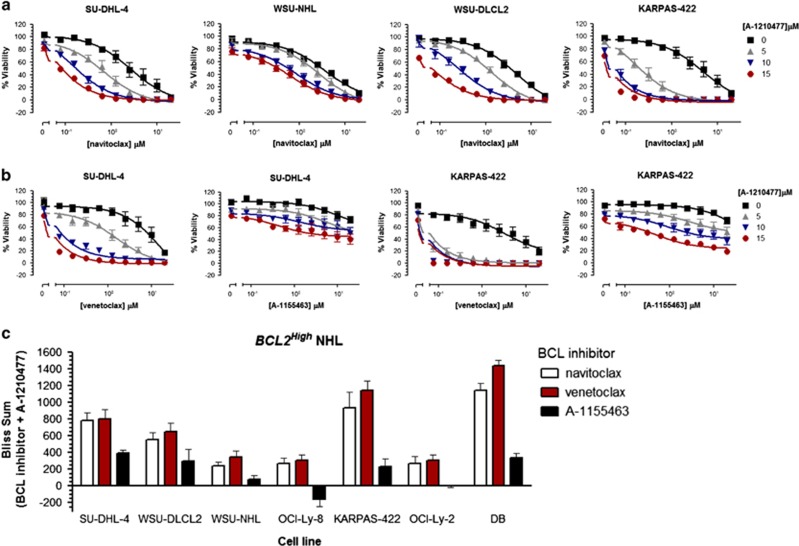
The MCL-1 inhibitor A-1210477 synergizes with navitoclax in *BCL2*^*High*^ NHL cell lines via BCL-2 and not BCL-X_L_ inhibition. NHL *BCL2*^*High*^ cell lines were co-treated with navitoclax (0–20 μM); (**a**), the BCL-2-selective inhibitor venetoclax (0–20 μM) or the BCL-X_L_-selective inhibitor A-1155463 (0–20 μM); (**b**), in combination with the MCL-1-specific inhibitor A-1210477 (0, 5, 10 and 15 μM) for 48 h and the effect on viability determined. Synergy was quantified using the Bliss algorithm (**c**). Data are presented as the mean±s.e.m. of three independent experiments.

**Figure 3:**
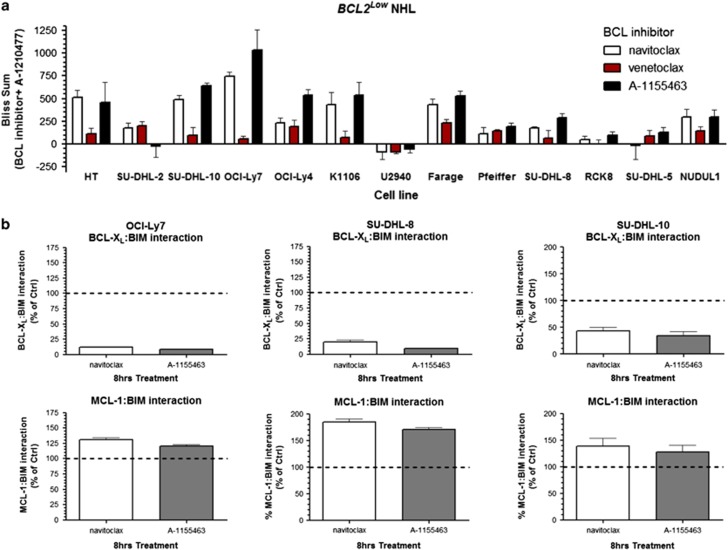
The MCL-1 inhibitor A-1210477 synergizes with navitoclax in *BCL2*^*Low*^ NHL cell lines via BCL-X_L_ and not BCL-2 inhibition. NHL *BCL2*^*Low*^ cells were treated as in Figure 2 and the degree of synergy determined by Bliss analysis (**a**). *BCL2*^*Low*^ NHL cell lines were treated with navitoclax or A-1155463 (all 1 μM) for 8 h and the interaction of BIM with BCL-X_L_ or MCL-1 assessed using an electrochemiluminescent ELISA (MSD) as described in the Materials and Methods section (**b**). Data are presented as the mean±s.e.m. of three independent experiments.

MCL-1 can be considered an intrinsic as well as an acquired resistance factor that limits the efficacy of navitoclax, ABT-737 and venetoclax.^24, 25, 26, 27, 28^ However, MCL-1 protein expression does not directly correlate with the sensitivity of NHL cell lines to navitoclax or venetoclax (Figure 1b and Supplementary Figure 2). Since some *BCL2*^*High*^ NHL cell lines are relatively resistant to navitoclax (EC_50_ >2 μM; Figure 1a and Supplementary Table 1), we treated *BCL2*^*High*^ NHL cell lines (SU-DHL-4, WSU-NHL and WSU-DLCL2) with navitoclax or venetoclax and evaluated interactions of BIM with BCL-2 and MCL-1. Both navitoclax and venetoclax disrupted BCL-2:BIM interactions; however, this was accompanied by an enhanced association of BIM with MCL-1 (Figure 1c). We hypothesize that MCL-1 functions as a sink in reserve that sequesters free BIM and prevents subsequent BAX activation. We therefore treated a panel of navitoclax-resistant *BCL2*^*High*^ cell lines with the recently described MCL-1-specific inhibitor A-1210477^(refs 28, 32)^ in combination with navitoclax. A-1210477 alone had minimal effect on cell viability but substantially sensitized resistant *BCL2*^*High*^ NHL cell lines to navitoclax (Figure 2a). To determine whether the synergy between navitoclax and A-1210477 was driven by BCL-2 or BCL-X_L_ inhibition, we treated these cell lines with the BCL-2-selective inhibitor venetoclax or the BCL-X_L_-selective inhibitor A-1155463 as adjuvants to A-1210477. A-1210477-sensitized *BCL2*^*High*^ NHL cells to venetoclax but not A-1155463 (Figure 2b and c). The synergy observed with A-1210477 was often greater than that obtained with navitoclax and A-1210477 (Figure 2c). Navitoclax and venetoclax but not A-1155463 also synergized with A-1210477 in more sensitive *BCL2*^*High*^ NHL cells such as DB (venetoclax EC_50_ is 166.5 nM) and OCI-Ly2 (venetoclax EC_50_ is 958.3 nM; Figure 2c). Conversely, chemical dissection of synergy between navitoclax and A-1210477 in *BCL2*^*Low*^ lines revealed cell death to be primarily driven by BCL-X_L_ inhibition (Figure 3a). Treatment of navitoclax-sensitive or resistant *BCL2*^*Low*^ NHL cell lines (SU-DHL-8, SU-DHL-10 and OCI-Ly-7) with navitoclax or A-1155463 resulted in dissociation of BIM from BCL-X_L_ and enhanced MCL-1:BIM interactions (Figure 3b).

**Figure 4:**
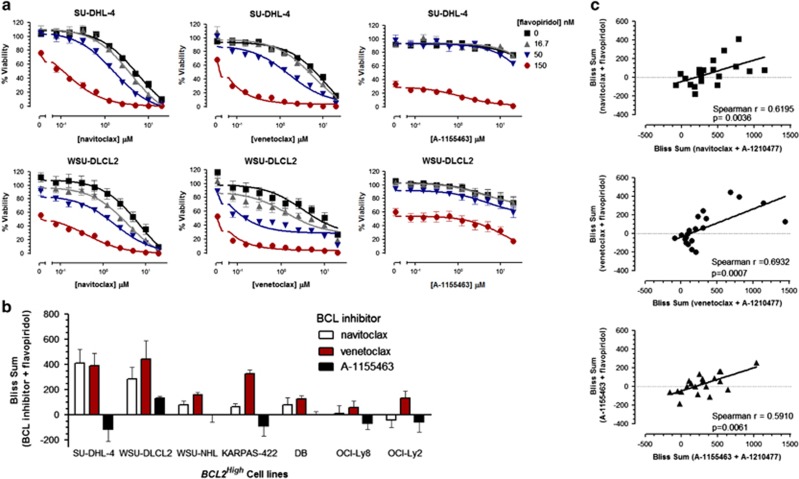
Synergy between A-1210477 and BCL-2 family inhibitors correlates with that observed between flavopiridol and BCL-2 family inhibitors. *BCL2*^*High*^ NHL cell lines were co-treated with navitoclax (0–20 μM), venetoclax (0–20 μM) or A-1155463 (0.20 μM) in combination with flavopiridol (0, 16.67, 50 and 150 nM) for 48 h and the effect on viability determined (**a**). Synergy was quantified by Bliss analysis (**b**). Data are presented as the mean±s.e.m. of three independent experiments. Bliss sums obtained in both *BCL2*^*High*^ and *BCL2*^*Low*^ NHL cell lines treated with A-1210477 in combination with navitoclax, venetoclax or A-1155463 were then correlated with those obtained with flavopiridol and navitoclax, venetoclax or A-1155463 (**c**). Data points represent the mean of three independent experiments. Spearman rank correlation co-efficient and associated statistical significance was determined using GraphPad Prism.

**Figure 5:**
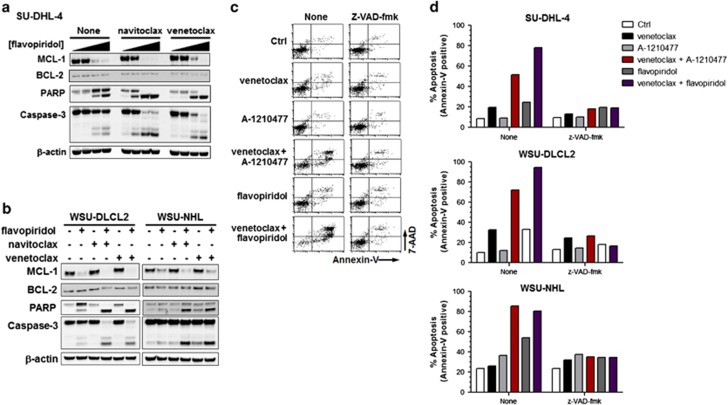
Flavopiridol-mediated downregulation of MCL-1 sensitizes *BCL2*^*High*^ NHL cell lines to navitoclax and venetoclax in a caspase-dependent manner. *BCL2*^*High*^ NHL cell lines were co-treated with navitoclax (1 μM) or venetoclax (1 μM) in combination with flavopiridol at 0, 16.67, 50 or 150 nM (**a**) or 50 nM (**b**) for 24 h before assessing effects on MCL-1, BCL-2, caspase-3, PARP and β-actin by western blot. Alternatively *BCL2*^*High*^ cell lines were pre-treated with z-VAD-fmk (50 μM) for 1 h and then co-treated with navitoclax (1 μM) or venetoclax (1 μM) in combination with A-1210477 (5 μM) or flavopiridol (50 nM) for a further 24 h and the effect on apoptosis determined by flow cytometric analysis of Annexin-V/7-AAD staining. Representative flow cytometry histograms in SU-DHL-4 cells from three independent experiments are shown in **c** and quantified in **d**.

The CDK inhibitor flavopiridol has been assessed clinically in NHL patients^37, 38, 39, 40^ and is a transcriptional repressor of MCL-1 expression.^41, 42^ We have demonstrated that A-1210477 induces a cellular phenotype similar to that observed following repression of MCL-1 expression through inhibition of transcriptional elongation by flavopiridol.^27^ Flavopiridol therefore represents a clinically relevant surrogate for inhibiting MCL-1 function. Similarly to A-1210477, flavopiridol co-treatment sensitized *BCL2*^*High*^ NHL cell lines to navitoclax- or venetoclax-induced cell death but not A-1155463 in a synergistic manner (Figure 4a and b). In fact, synergy between navitoclax, venetoclax or A-1155463 and A-1210477 was predictive of synergy with flavopiridol in all NHL cell lines analyzed (Figure 4c). Flavopiridol reduced the expression of MCL-1 and, in combination with navitoclax or venetoclax, resulted in enhanced PARP cleavage and caspase-activation indicating an apoptotic phenotype (Figure 5a and b). To further validate the mechanism of cell death and synergy, we evaluated annexin-v/7-AAD staining by flow cytometry in the presence or absence of the broad spectrum caspase inhibitor z-VAD-fmk. A-1210477 potentiated the degree of annexin-v/7-AAD staining induced by either navitoclax or venetoclax. This staining was inhibited by z-VAD-fmk, indicating that caspase activity is required for this apoptotic phenotype. Similarly, flavopiridol-sensitized *BCL2*^*High*^ NHL cells to venetoclax-mediated apoptosis as evidenced by high annexin-v/7-AAD staining that was caspase-dependent (Figure 5c and d).

## Discussion

Although navitoclax has shown encouraging activity in hematologic malignancies as a single agent and in the adjuvant setting, its clinical utility is limited by thrombocytopenia driven by inhibition of BCL-X_L_.^14, 15, 16, 17, 18, 19^ We recently described the development of the BCL-2-selective inhibitor venetoclax that shows superior affinity for BCL-2 and excellent selectivity over BCL-X_L_. This translates into increased potency and efficacy in pre-clinical models of lymphoid malignancies that are dependent on BCL-2 for survival.^20^ Importantly, its lack of affinity for BCL-X_L_ circumvents thrombocytopenia, a dose-limiting toxicity associated with navitoclax.^20^ Subsequently, objective responses have been obtained in clinical trials of venetoclax in chronic lymphocytic leukemia^43^ and NHL^44^ patients. MCL-1 and BCL-X_L_ are intrinsic and acquired resistance factors that limit the efficacy of navitoclax or ABT-737^(refs 25, 26, 27, 28, 29)^ and therefore may impact the clinical utility of venetoclax. We therefore sought to understand the functional roles of MCl-1 and BCL-X_L_ in NHL cell lines with intrinsic resistance to venetoclax.

We have segregated NHL cell lines into two populations; *BCL2*^*High*^ represents lines with high *BCL2* CN and/or the *BCL2* translocation t(14;18), whereas lines without these lesions were defined as *BCL2*^*Low*^. As a population, *BCL2*^*High*^ NHL cell lines are largely sensitive to navitoclax- or venetoclax-induced apoptosis. Despite this, some *BCL2*^*High*^ NHL cell lines are relatively resistant to venetoclax, with *EC*_*50*_s >2 μM *in vitro*, a facet we hypothesized here to be a consequence of MCL-1 function and not simply expression. Expression of MCL-1 at the protein level does not directly correlate with resistance to navitoclax or venetoclax in NHL cell lines herein, or at the gene level with ABT-737 in chronic lymphocytic leukemia.^24^ Treatment of resistant NHL cell lines with navitoclax, venetoclax or A-1155463 resulted in enhanced MCL-1:BIM interactions that we hypothesized to inhibit BAX/BAK activation and subsequently limit the efficacy of these compounds. This capacity of MCL-1 to function as a ‘sink' for additional free or displaced BIM serves as a survival response to cellular stress mediated by BCL-2 and/or BCL-X_L_ inhibition. However, this process also primes these cells for death by agents that inhibit MCL-1 function.

Loss of MCL-1 function through gene silencing or indirect pharmacological inhibition sensitizes many tumor types to navitoclax.^24, 25, 26, 27, 28^ A-1210477 is a MCL-1-specific inhibitor that induces apoptosis in a phenotypically identical fashion to MCL-1 siRNA.^27, 28, 32^ Herein, we have used A-1210477 and other selective BCL-2 family inhibitors to define the contributions of MCL-1, BCL-2 and BCL-X_L_ in maintaining the survival of various NHL cell lines. These ‘chemical parsing' experiments^45^ demonstrated that sensitization of *BCL2*^*High*^ NHL cell lines to navitoclax in response to direct MCL-1 inhibition with A-1210477 or indirectly through loss in MCL-1 expression mediated by flavopiridol, is driven by BCL-2 inhibition with no contribution from BCL-X_L_. The BCL-2-selective inhibitor venetoclax was equivalent, if not slightly superior, to navitoclax in inducing caspase-dependent cell death in synergy with A-1210477. The BCL-X_L_-selective inhibitor A-1155463 showed no single agent activity and did not synergize with A-1210477 in *BCL2*^*High*^ NHL cells *in vitro*, further emphasizing the importance of BCL-2 inhibition in the *BCL2*^*High*^ subtype.

Synergy between A-1210477 and navitoclax, venetoclax or A-1155463 correlated with that observed with flavopiridol (Figure 4). However, the degree of synergy observed with flavopiridol and venetoclax or navitoclax is less than that observed with A-1210477; perhaps because loss in MCL-1 function achieved through cellular exposure to A-1210477 is mechanistically distinct from that of flavopiridol. A-1210477 binds to the BH3-binding groove of MCL-1 and results in stabilization of MCL-1 protein levels.^28, 32^ This is analogous to that observed with BIM BH3 peptides.^46^ In contrast, flavopiridol treatment results in a loss in MCL-1 protein expression (Figure 5a and b) through transcriptional repression of MCL-1.^41, 42^ However, CDK9 regulates several other genes that dictate cellular survival,^47^ and flavopiridol's effect on their expression may also contribute to synergy with navitoclax, venetoclax or A-1155463.

Despite their *BCL2*^*Low*^ NHL classification, SU-DHL-8 and RCK8 cell lines are sensitive to navitoclax. In this case, chemical parsing^45^ experiments revealed that this efficacy was driven by BCL-X_L_ inhibition since the BCL-X_L_-selective inhibitor A-1155463 was efficacious, whereas the BCL-2-selective inhibitor venetoclax was not. These data are in contrast to a recent finding by Merino *et al.*,^29^ who proposed that navitoclax does not bind to BCL-X_L_ with sufficient avidity to kill lymphoid cells efficiently. Indeed, we found that navitoclax was able to significantly perturb the BCL-X_L_:BIM interactions (Figure 3b). The dependency of SU-DHL-8 and RCK8 on BCL-X_L_ for survival is further exemplified at the protein and gene level. These cell lines are characterized as possessing low BCL-2 protein levels (Figure 1b) and high *BCL-X*_*L*_ (*BCL2L1*) CN (Supplementary Table 1). Furthermore, apoptosis and synergy between navitoclax and the MCL-1 inhibitor A-1210477 in *BCL2*^*Low*^ cell lines generally required BCL-X_L_ inhibition and not BCL-2. We speculate that the *BCL2*^*Low*^ characterization may therefore represent a NHL patient population that may benefit from navitoclax rather than venetoclax treatment in the combination setting, such as with bendamustine/rituximab.^20, 48^

Taken together, the data described herein demonstrate that approaches to inhibit MCL-1 function can be combined with venetoclax, a selective BCL-2 inhibitor, in pre-clinical models of NHL. Combined treatment of *BCL2*^*High*^ NHL cell lines with venetoclax and A-1210477 or flavopiridol results in the synergistic induction of apoptosis *in vitro*. Importantly, the BCL-X_L_-selective inhibitor A-1155463 is not efficacious as a single agent or in combination with MCL-1 inhibitors in *BCL2*^*High*^ NHL cell lines *in vitro*. Collectively these data emphasize that *BCL2* status is predictive of venetoclax efficacy in NHL not only as a single agent, but also in the adjuvant setting with anti-tumorigenic agents that modulate MCL-1 levels. Finally, we demonstrate that the *BCL2*^*Low*^ NHL classification predicts navitoclax combinational efficacy due to a requirement for BCL-X_L_ inhibition and not BCL-2. Elevated levels of MCL-1 have been described in chronic lymphocytic leukemia, MCL and multiple myeloma^49, 50, 51, 52, 53^ and this study in pre-clinical models of NHL paves the way to evaluate the consequence of functional inhibition of MCL-1 in combination with venetoclax in these additional hematologic malignancies.
