# Supplementary figures and images for: Loss in MCL-1 function sensitizes non-Hodgkin's lymphoma cell lines to the BCL-2-selective inhibitor venetoclax (ABT-199)
Source: Blood Cancer J. 2015 Nov 13;5(11):e368–. doi: 10.1038/bcj.2015.88 (PMC4670945; doi:10.1038/bcj.2015.88)

## Slide 1
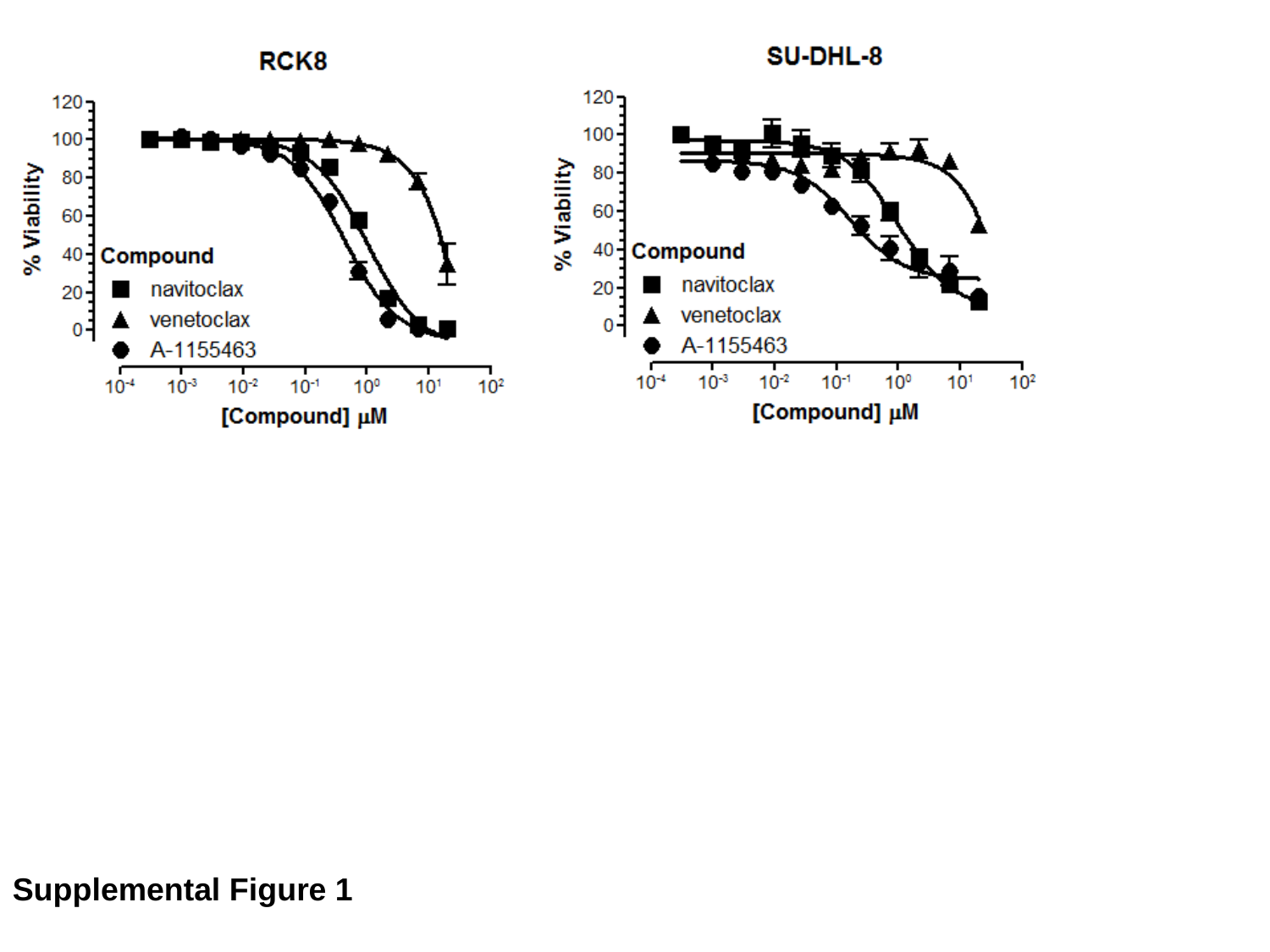

Supplemental Figure 1

Supplement: Supplementary Figure 1 [file bcj201588x1.ppt]

## Slide 1
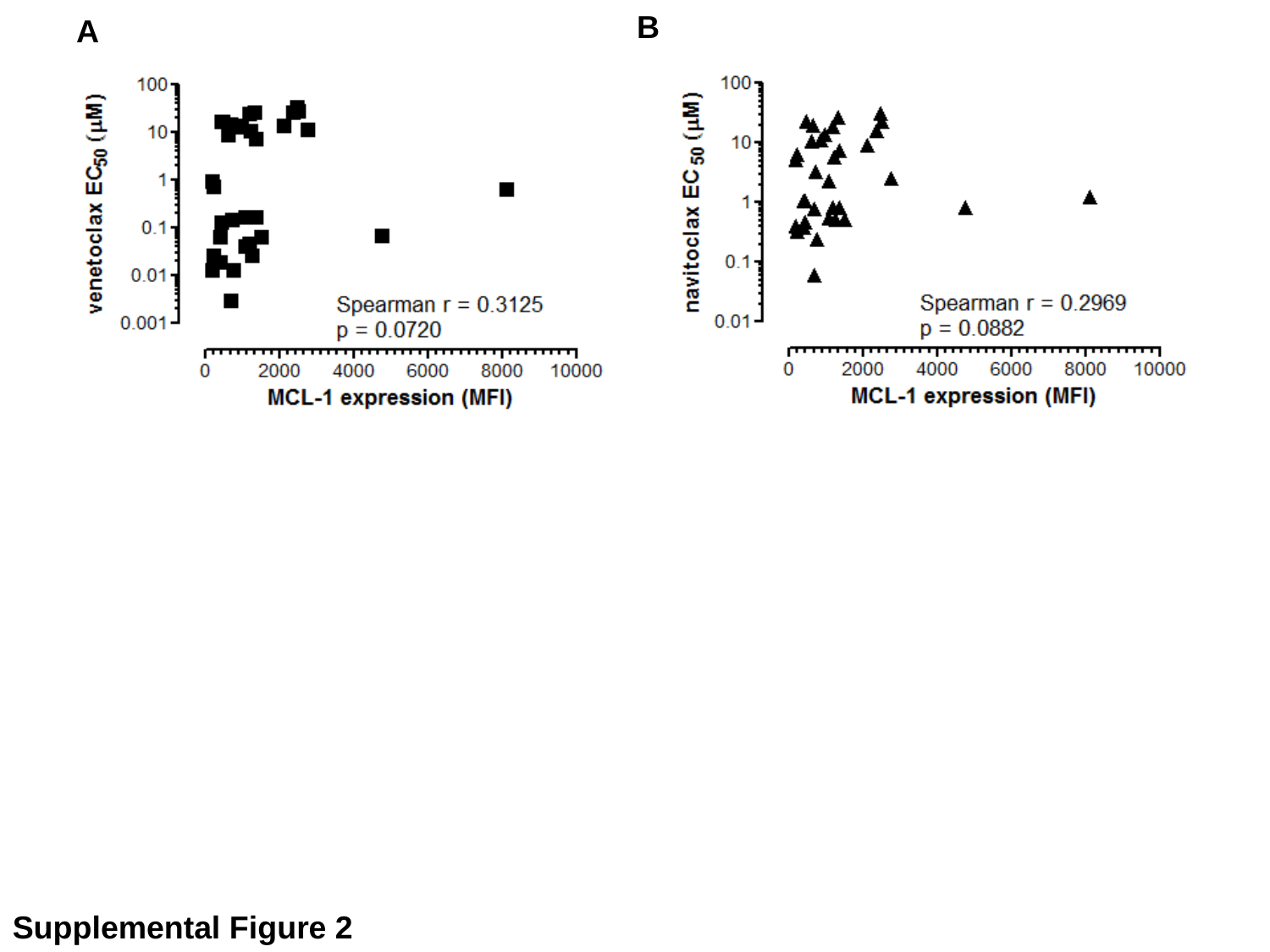

B
A
Supplemental Figure 2

Supplement: Supplementary Figure 2 [file bcj201588x2.ppt]

## Slide 1
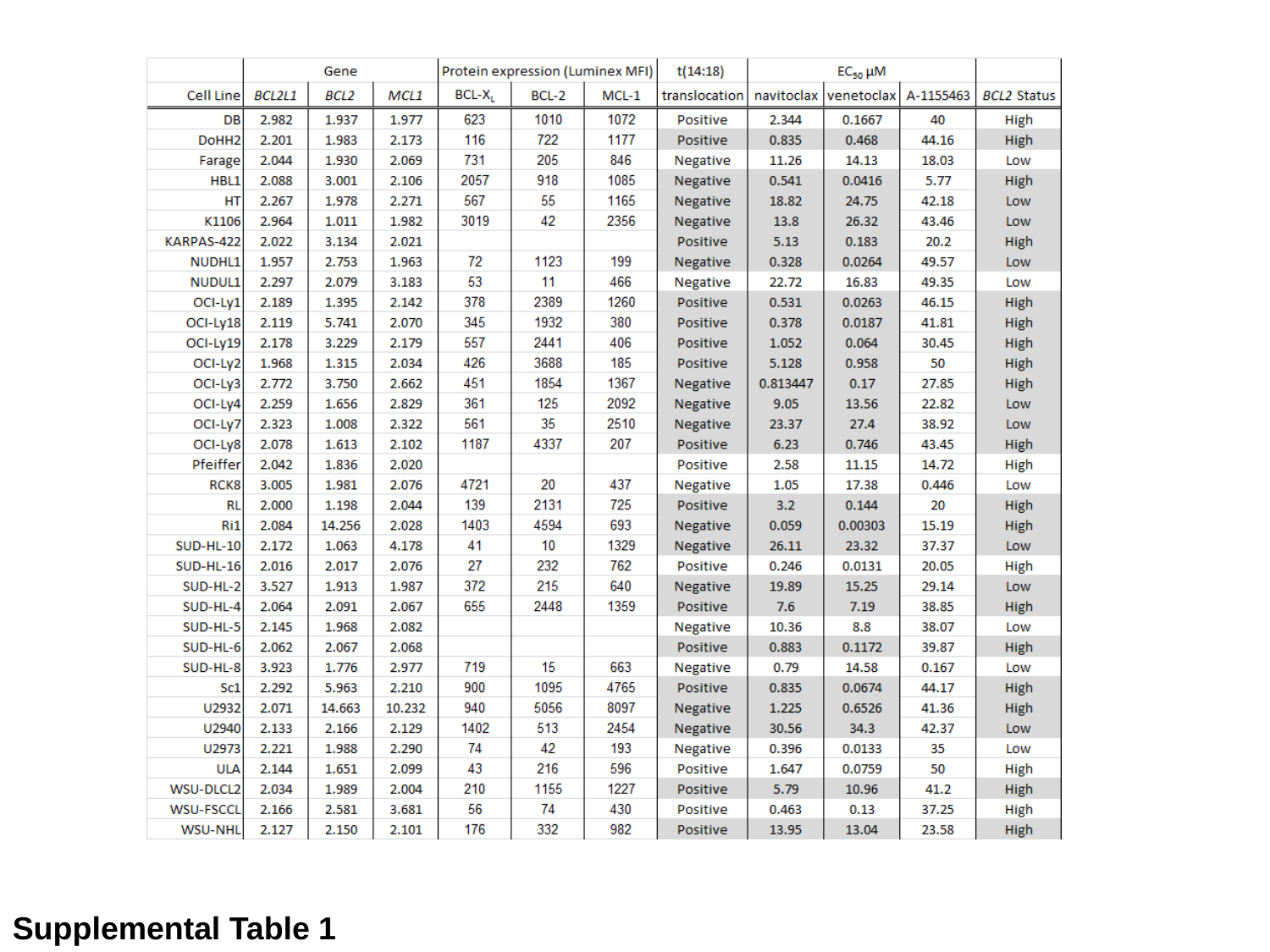

Supplemental Table 1

Supplement: Supplementary Table 1 [file bcj201588x3.ppt]
