# Supplementary material for: Loss in MCL-1 function sensitizes non-Hodgkin's lymphoma cell lines to the BCL-2-selective inhibitor venetoclax (ABT-199)
Source: Blood Cancer J. 2015 Nov 13;5(11):e368–. doi: 10.1038/bcj.2015.88 (PMC4670945; doi:10.1038/bcj.2015.88)
Supplement: Supplementary Information [file bcj201588x4.doc]

**Supplemental Figure 1. *BCL2^Low^* NHL cell lines RCK-8 and SU-DHL-8 are dependent upon BCL-X_L_ for survival**. RCK-8 and SU-DHL-8 cells were treated with navitoclax, venetoclax or A-1155463 for 48hrs in media containing 10% human serum and cell viability determined using CellTiter-Glo™. Data are presented as the mean ± s.e.m. of three independent experiments.

**Supplemental Figure 2. Venetoclax or navitoclax efficacy does not correlate with MCL-1 protein expression in NHL cell lines.** MCL-1 protein expression as determined in Figure 1B was correlated with venetoclax (A) or navitoclax (B) EC_50_ as determined in Figure 1a. Spearman rank correlation co-efficient and associated statistical significance was determined using GraphPad Prism.

**Supplemental Table 1. Amplification and protein expression of the anti-apoptotic BCL-2 family members, t(14;18) translocation status in NHL cell lines and cellular potencies associated with BCL-2 family inhibitors.** Data were determined as described in the Materials and Methods. Note that EC_50_s were determined in media containing 10% human serum (HS) following a 48 hr treatment period and represent the mean of 3-14 independent experiments. Grey shading represents data previously reported by .
